# A Robust Indoor/Outdoor Navigation Filter Fusing Data from Vision and Magneto-Inertial Measurement Unit

**DOI:** 10.3390/s17122795

**Published:** 2017-12-04

**Authors:** David Caruso, Alexandre Eudes, Martial Sanfourche, David Vissière, Guy Le Besnerais

**Affiliations:** 1 Computer Vision R and D department, Sysnav, 57 rue de Montigny, 27200 Vernon, France; david.vissiere@sysnav.fr; 2 Department of Information Processing and Systems, ONERA, the French Aerospace Lab, Chemin de la Hunière, 91120 Palaiseau, France; alexandres.eudes@onera.fr (A.E.); martial.sanfourche@onera.fr (M.S.); guy.le_besnerais@onera.fr (G.L.B.)

**Keywords:** Visual-inertial Navigation Systems (VINS), Magneto-inertial Dead reckoning (MI-DR), vision-based navigation, IMU navigation, MSCKF, error-state kalman filter

## Abstract

Visual-inertial Navigation Systems (VINS) are nowadays used for robotic or augmented reality applications. They aim to compute the motion of the robot or the pedestrian in an environment that is unknown and does not have specific localization infrastructure. Because of the low quality of inertial sensors that can be used reasonably for these two applications, state of the art VINS rely heavily on the visual information to correct at high frequency the drift of inertial sensors integration. These methods struggle when environment does not provide usable visual features, such than in low-light of texture-less areas. In the last few years, some work have been focused on using an array of magnetometers to exploit opportunistic stationary magnetic disturbances available indoor in order to deduce a velocity. This led to Magneto-inertial Dead-reckoning (MI-DR) systems that show interesting performance in their nominal conditions, even if they can be defeated when the local magnetic gradient is too low, for example outdoor. We propose in this work to fuse the information from a monocular camera with the MI-DR technique to increase the robustness of both traditional VINS and MI-DR itself. We use an inverse square root filter inspired by the MSCKF algorithm and describe its structure thoroughly in this paper. We show navigation results on a real dataset captured by a sensor fusing a commercial-grade camera with our custom MIMU (Magneto-inertial Measurment Unit) sensor. The fused estimate demonstrates higher robustness compared to pure VINS estimate, specially in areas where vision is non informative. These results could ultimately increase the working domain of mobile augmented reality systems.

## 1. Introduction

### 1.1. Motivation

Infrastructure-less navigation and positioning in indoor location is a technical prerequisite for numerous industrial and consumer applications: ranging from lone worker safety in industrial facilities, to augmented reality. Still, it remains an open challenge to efficiently and reliably combine embedded sensors to reconstruct a position or a trajectory. In the present work, we address this challenge restricting ourselves to the following sensors: MEMS gyroscopes, accelerometers, magnetometers and a standard industrial vision camera. We motivate this choice by the fact these sensors are cheap and can easily be embedded in a wearable form factor, which makes this combination appealing for pedestrian application. Moreover, VINS (Visual-Inertial Navigation Systems) literature, showed recently tremendous progress in the past few years.

### 1.2. State of the Art and Contribution

Indeed, if a wide range of embedded visual sensors were previously presented to solve the problem, such as rotating LIDARs [1] or depth sensors [2], much of the recent efforts focused on conventional cameras, as they are cheap, and already present in a wide range of lightweight devices, such as smart-phones. While authors of [3,4,5] rely on multiple embedded cameras, single cameras solutions have been shown to provide good results. For instance, authors of [6,7] present efficient filtering methods for monocular VINS. Nonetheless, monocular VINS remains highly sensitive to degenerate motions scenarios. For instance, the scale factor is weakly constrained in case of a steady motion and pure rotation motions must be tackled with special care in the estimation process. Moreover, current VINS implementations rely heavily on high frequency visual corrections (10–20 Hz), and break when visual environment is not adequate for more than a few second (bad illumination, presence of smoke, motion blur, etc.).

Compared to the previous literature, we explore a sensor suite alternative to VINS and able to provide a precise and robust navigation information. We combine the vision sensor with a MIMU (Magneto-Inertial Measurement Unit), i.e., an IMU sensor augmented with *an array of* magnetometers. Within a stationary and non-uniform magnetic environment, the magnetic measurements render the body speed observable, which has been shown to significantly improve motion prediction compared to IMU alone [8,9]. This technique, named Magneto-Inertial Dead-Reckoning (MI-DR) hereafter, is perfectly fitted for indoor navigation as human made environment, wall, floor, and furnitures all perturb the magnetic field in a significant way. As a result, the open-loop position error of this method is often around a few percent of the trajectory length in indoor environment (see [10]). MI-DR fails however in places where the gradient of the magnetic field vanishes, (commonly outdoor) and lacks robustness when the magnetic environment is not stationary.

We have already presented various approaches to fuse the information from MIMU sensor with vision sensors for dead-reckoning estimation. In [11], we proposed a semi-tight fusion scheme combining a depth sensor with a MIMU to increase robustness and availability of the position/orientation informations. Yet, we finally turned towards conventional monocular camera, mainly because the limited range of depth sensors makes them unable to improve the MI-DR estimation in large rooms or outdoor. In order to still be able to estimate accurately the scale, we also turned towards a fully tight fusion scheme, that includes estimation of camera pose, current speed, magnetic field and inertial sensor biases. We investigated an optimization-based solution in [12] and a filter-based solution in [13]. Here we present an extension of the work presented in the conference paper [13] with new results and a slightly different implementation of the filter. The used estimator is still inspired by the pure VINS method presented in [14] for the visual measurements and by the magnetic prediction and measurement process of [9] for the MIMU handling process. We will call it MI-MSCKF for Magneto-inertial Multi State Constraint Kalman Filter. As in [13], we choose a square-root implementation which leads to a better overall conditioning of matrices operations involved in the filtering process. The inverse form is also computationally interesting for high-dimensionality measurements [15] (p. 141).

We show on experimental data that the MIMU provides robustness in situations where vision fails, and that, reciprocally, vision allows the system to handle situations where the magnetic gradient is to low for the MIMU to work properly, typically in outdoor situations.

### 1.3. Paper Organization

After introducing general notations and conventions in Section 2, we describe the dynamic model our filter is built on in Section 3, with a focus on the magnetic prediction equation, less known in visual-inertial literature. This section also presents the model discretization and error model of the sensors. The Section 4 describes the filter design: chosen state and equations for propagation and measurement update steps. The last section (Section 5) presents comparative results of trajectory estimation obtained on real datasets.

## 2. Notations

### 2.1. General Conventions

Bold capital letters X denote matrices or elements of manifold. Parenthesis are used to denote the Cartesian product of two elements a∈A,b∈B↦(a,b)∈A×B and brackets for the concatenation of two row vectors. For a vector $x={\left[x_{1},x_{2},x_{3}\right]}^{T}$, $x_{2}$ denotes its second component $x_{2}$ and $x_{2:3}$ is the sub-vector ${\left[x_{2},x_{3}\right]}^{T}$. For matrices, we define the vectorization operation $\mathrm{Vec}\left(\right)$ so that:

(1) $$\mathrm{Vec}\left(\left[\begin{array}{cc} x_{11} & x_{12}\\ x_{21} & x_{22} \end{array}\right]\right)={\left[\begin{array}{c} x_{11},x_{21},x_{12},x_{22} \end{array}\right]}^{T}.$$

A⊗B will denotes the Kronecker product of matrices A and B. $\partial_{A}$ will be a shorthand for the derivative with respect to the coefficients of $\mathrm{Vec}\left(A\right)$. The notation ${∥x∥}_{\Sigma }$ is the Mahalanobis norm of invertible covariance Σ: ${∥x∥}_{\Sigma }=x^{T}\Sigma^{-1}x$. $I_{n}$ is the identity matrix of size *n* and $0_{n\times m}$ the zero matrix of size n×m. $I_{n}$ is the identity matrix of size n×n or corresponding application. O(n) is the orthogonal matrix group of size n.

### 2.2. Reserved Symbols

**Figure 1:**
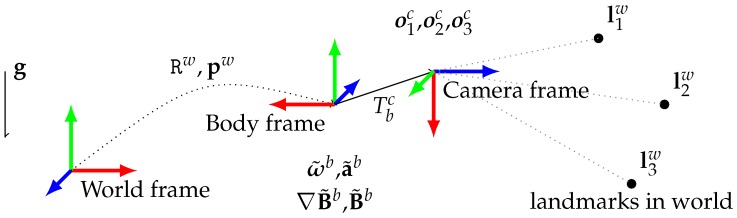
Reference coordinate frames at play in the problem, with associated typical measurements.

Generally and except stated otherwise we use the following symbols: p for the translational part of the body pose, R for its rotational part. v for the velocity, $b_{a}$ and $b_{g}$ for inertial sensor bias, B for the magnetic field, ∇B for its 3×3 gradient matrix. ω is used for the rotational speed and a for the specific acceleration. 3D landmark position are noted with the letter l and these observation into an image with the letter o. We use a tilde symbol for measured quantities or generally quantities that can be derived from sensor reading. We use an hat for estimated version of a physical quantity. g symbol is kept for the gravity vector in inertial coordinates. The world coordinates are defined such that the gravity vector writes $g≃{\left[0,0,-9.81\right]}^{T}$. When ambiguous, the reference frame in which a quantity is expressed will be noted in exponent: w stands for the world gravity-aligned reference frame, b for the current body reference frame and c for the camera frame. The Figure 1 summarizes the chosen notations.

### 2.3. Rotation Parametrization

For rotations, we use the convention that $R^{w}$ transforms a vector from body frame to world frame by left multiplying it. For the sake of clarity of the developments, we represent the attitude of the sensor as a rotation matrix. The Special Orthogonal Group is denoted SO(3) and its associated Lie algebra so(3)—the set of skew symmetric matrices. Any element of so(3) can be identified with a vector of $R^{3}$: ${\left[x\right]}_{\times }\in \mathrm{so}\left(3\right)$ with $x\in R^{3}$ and vex so that $vex({\left[x\right]}_{\times })=x$. exp and log are the standard exponential map and logarithm on SO(3). As in [16], we use vectorized versions of exp and log:

(2) $$\begin{array}{c} \mathrm{Exp}:\begin{array}{ccc} R^{3} & \to & \mathrm{SO}(3)\\ \delta \theta & \mapsto & \exp({\left[\delta \theta \right]}_{\times }) \end{array} \end{array}$$

and $\mathrm{Log}:\mathrm{SO}\left(3\right)\to R^{3}$ the inverse function. With these conventions, Log(Exp(x))=x.

## 3. On-Board Sensors and Evolution Model

### 3.1. Sensing Hardware

**Figure 2:**
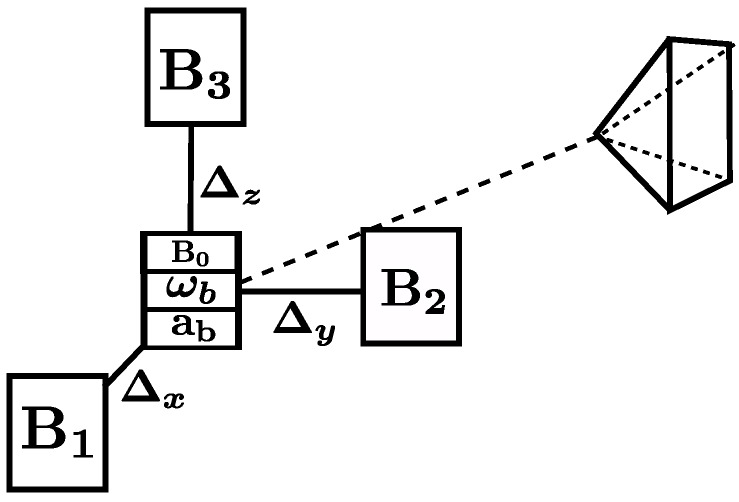
Schematic view of on-board sensors. In addition to accelerometers and gyrometers, the MIMU includes several magnetometers: a central one and at least three peripheral ones in order to compute the full 3×3 matrix of magnetic field gradients. The camera is rigidly attached to the MIMU sensor.

The MIMU sensors (see Figure 2) provide raw measurements of biased proper acceleration ${\tilde{a}}_{}^{b}$, biased angular velocity ${\tilde{\omega }}_{}^{b}$, magnetic field ${\tilde{B}}_{}^{b}$ and its gradients $\nabla {\tilde{B}}_{}^{b}$. The latter is a 3×3 matrix which elements are estimated by finite differences between signals recorded on an array of magnetometers. These sensors are carefully calibrated offline and harmonised in the same spatial coordinate frame with a method similar to [17]. The resulting MIMU coordinate frame, centered around the magnetometer and accelerometer (which are assumed colocalized here), will be used as the *body* frame in all subsequent derivations. We use a global shutter camera modeled as a pinhole camera with instantaneous exposure. In practice, recorded real images are undistorted using intrinsics calibration parameters. Intrinsic parameters (focal length in pixels $\left(f_{x},f_{y}\right)$, principal point coordinates $\left(c_{x},c_{y}\right)$ and distortion coefficients) are assumed to be known from a preliminary calibration. The pinhole camera projection function π maps a landmark 3D coordinates $l^{c}$ expressed in the camera frame to the pixel coordinates of its projection onto the image:

(3) $$\begin{array}{c} \pi :\begin{array}{ccc} R^{3} & \to & R^{2}\\ l^{c} & \mapsto & \left[\begin{array}{c} f_{x}\frac{l_{1}^{c}}{l_{3}^{c}}+c_{x}\\ f_{y}\frac{l_{2}^{c}}{l_{3}^{c}}+c_{y} \end{array}\right] \end{array}. \end{array}$$

The camera is rigidly attached to the MIMU sensor board. The transformation $\left(R_{c}^{b},p_{c}^{b}\right)$ between the camera frame and the body frame is assumed to be known. We could alternatively include this transform into the state filter as done in [18] for instance.

Since hardware synchronization was not possible with the components used here, we use a datation approach. In the online estimation process we make the simplifying assumption that images are captured simultaneously with the MIMU sample closest in time. More precisely, the image information at time $t_{{image}_{p}}$ is processed using the state estimate at time $t_{{mimu}_{k}}$ with *k* such as:

$$k={\arg\;\min}_{k,t_{{mimu}_{k}}<t_{{image}_{p}}}\left|t_{{mimu}_{k}}-t_{{image}_{p}}\right|.$$

The temporal error done with this approach is always smaller than the sampling period of the MIMU sensors, which is often below the exposure time of the camera. Besides, this approximation significantly simplifies time management as all measurements are indexed by the MIMU time sampling.

### 3.2. Evolution Model

In order to estimate the position and orientation of the body coordinate frame in the world frame, one has to track an estimate of its speed and of the magnetic field at the current position of its center. We model the evolution of these quantities with the following differential equations:

(4) $$\begin{array}{cc} {\dot{R}}^{w}\left(t\right) & =R^{w}\left(t\right){\left[\omega^{b}\left(t\right)\right]}_{\times }, \end{array}$$

(5) $$\begin{array}{cc} {\dot{p}}^{w}\left(t\right) & =R^{w}\left(t\right)v^{b}\left(t\right), \end{array}$$

(6) $$\begin{array}{cc} {\dot{v}}^{b}\left(t\right) & =-{\left[\omega^{b}\left(t\right)\right]}_{\times }v^{b}\left(t\right)+R^{wT}\left(t\right)g^{w}+a^{b}\left(t\right), \end{array}$$

(7) $$\begin{array}{cc} {\dot{B}}^{b}\left(t\right) & =-{\left[\omega^{b}\left(t\right)\right]}_{\times }B^{b}\left(t\right)+\nabla B^{b}\left(t\right)v^{b}\left(t\right). \end{array}$$

This model relies on the following assumptions on the environment:

- **Flat-earth approximation.** We assume that the Est-North-Up (ENU) earth frame at filter initialization is an inertial frame.
- **Stationary magnetic field** in the world frame—although possibly spatially non-uniform, leading to the spatial gradient in (7).

The first assumption is, in practice, often used in VINS literature, in which gyroscopes are not precise enough to measure earth rotational speed (roughly $7\times 10^{-5}\;\mathrm{rad}/s$). However if high-end gyroscopes had been used, it is likely that an estimate based on the simple model (4)–(7) would introduce some error, confusing earth rotational velocity with biases estimates. In our opinion though, even in the case of high-end hardware, it is not clear that the visual pipeline used in this work would be able to provide information reliable enough to estimate biases with sufficient accuracy to be influenced by earth rotational speed. This would be a great challenge to address.

Note also that the world frame differs from the ENU frame defined at filter initialization: they both have their origin at the position of the center of the body frame at initial time, but they can differ by a rotation around the gravity direction, as heading is not observable at initialization.

Equation (7) is the key equation for MI-DR. It relates the evolution of the magnetic field with kinematics quantities and local magnetic gradient. It actually renders the body velocity observable provided the matrix $\nabla B^{b}$ is invertible. However, it fails giving useful translational information if the magnetic field is uniform. This happens outdoor, where the magnetic field is uniformly equal to the earth magnetic field or in very large empty rooms. In the latter situation, magnetic gradients can vanish a few meters away from a wall, ceiling or floor.

Also, the stationarity assumption can be challenged in some environments, or punctually if pieces of metal are moving in the vicinity of the magnetometers. While some instationarities can be modeled—as the case of power-line interference [9]—in practice, any useful estimator should also be robust to non modeled effects.

### 3.3. Model Discretization

The model will be used in a discrete extended Kalman filtering framework described in Section 4 below. We thus discretize it the following way:

(8) $$\begin{array}{cc} R_{k+1}^{w} & =R_{k}^{w}{\tilde{\Delta R}}_{kk+1}, \end{array}$$

(9) $$\begin{array}{cc} p_{k+1}^{w} & =p_{k}^{w}+R_{k}^{w}v_{k}^{b}\Delta t+\frac{1}{2}g^{w}\Delta t_{ij}^{2}+R_{k}^{w}{\tilde{\Delta p}}_{kk+1}, \end{array}$$

(10) $$\begin{array}{cc} v_{k+1}^{b} & ={\tilde{\Delta R}}_{kk+1}^{T}\left(v_{k}^{b}+{R_{k}^{w}}^{T}g^{w}\Delta t+{\tilde{\Delta v}}_{kk+1}\right), \end{array}$$

(11) $$\begin{array}{cc} B_{k+1}^{b} & ={\tilde{\Delta R}}_{kk+1}^{T}\left(B_{k}^{b}+{\tilde{D}}_{v;kk+1}v_{k}^{b}+{\tilde{D}}_{R;kk+1}{R_{k}^{w}}^{T}g^{w}+{\tilde{M}}_{kk+1}\right). \end{array}$$

where we introduced the following notation corresponding to continuous integrals:

(12) $$\begin{array}{cc} {\tilde{\Delta R}}_{kk+1} & \overset{\mathrm{def}}{=}\Delta R_{k}\left(t_{k+1}\right), \end{array}$$

(13) $$\begin{array}{cc} {\tilde{\Delta v}}_{kk+1} & \overset{\mathrm{def}}{=}{\tilde{\Delta v}}_{k}\left(t_{k+1}\right), \end{array}$$

(14) $$\begin{array}{cc} {\tilde{\Delta p}}_{kk+1} & \overset{\mathrm{def}}{=}\int_{t_{k}}^{t_{k+1}}{\tilde{\Delta v}}_{k}\left(\tau \right)d\tau , \end{array}$$

(15) $$\begin{array}{cc} {\tilde{D}}_{v;kk+1} & \overset{\mathrm{def}}{=}\int_{t_{k}}^{t_{k+1}}\Delta R_{k}\left(\tau \right)\nabla B^{b}\left(\tau \right)\Delta R_{k}{\left(\tau \right)}^{T}d\tau , \end{array}$$

(16) $$\begin{array}{cc} {\tilde{D}}_{R;kk+1} & \overset{\mathrm{def}}{=}\int_{t_{k}}^{t_{k+1}}\Delta R_{k}\left(\tau \right)\nabla B^{b}\left(\tau \right)\Delta R_{k}{\left(\tau \right)}^{T}\left[\tau -t_{k}\right]d\tau , \end{array}$$

(17) $$\begin{array}{cc} {\tilde{M}}_{kk+1} & \overset{\mathrm{def}}{=}\int_{t_{k}}^{t_{k+1}}\Delta R_{k}\left(\tau \right)\nabla B^{b}\left(\tau \right)\Delta R_{k}{\left(\tau \right)}^{T}\Delta {\tilde{v}}_{k}\left(\tau \right)d\tau , \end{array}$$

(18) $$\begin{array}{ccccc} \mathrm{with}\;\Delta R_{k}\left(\tau \right) & \overset{\mathrm{def}}{=}I_{3}+\int_{t_{k}}^{\tau }\Delta R_{k}\left(s\right){\left[\omega^{b}\left(s\right)\right]}_{\times }ds & \mathrm{and}\;\; & {\tilde{\Delta v}}_{k}\left(\tau \right) & \overset{\mathrm{def}}{=}\int_{t_{k}}^{\tau }\Delta R_{k}\left(s\right)a^{b}\left(s\right)ds. \end{array}$$

The notation $\Delta R_{k}\left(\tau \right)$ is the rotation matrix that transforms point in body frame at time τ to corresponding point in body frame at time $t_{k}$, that can be deduced from gyroscopes integration.

These integrals can be computed from unbiased MIMU measurements. We thus estimate the biases of the accelerometers and gyrometers along with previously quantities. These are assumed to follow a first-order Gauss-Markov stochastic evolution:

(19) $$\begin{array}{cc} \dot{b_{g}}\left(t\right) & =-\frac{1}{\tau_{g}}b_{g}+J_{bg}, \end{array}$$

(20) $$\begin{array}{cc} \dot{b_{a}}\left(t\right) & =-\frac{1}{\tau_{a}}b_{a}+J_{ba}. \end{array}$$

where generating noises $J_{bg}$ and $J_{ba}$ satisfy:

(21) $$\begin{array}{cc} E\left([J_{bg}\left(t\right),J_{ba}\left(t\right)]\right) & =0_{6\times 1}, \end{array}$$

(22) $$\begin{array}{cc} E\left(\left[J_{bg}\left(t_{1}\right),J_{ba}\left(t_{1}\right)\right].{\left[J_{bg}\left(t_{2}\right),J_{ba}\left(t_{2}\right)\right]}^{T}\right) & =W_{c}\delta \left(t_{2}-t_{1}\right), \end{array}$$

(23) $$W_{c}=\mathrm{diag}\left(\sigma_{bg;c}^{2}I_{3},\sigma_{ba;c}^{2}I_{3}\right).$$

$\tau_{b}$, $\tau_{a}$, $\sigma_{bg;c}$ and $\sigma_{ba;c}$ are expressed in *s*, *s*, $\frac{\mathrm{rad}}{s^{2}}\frac{1}{\sqrt{Hz}}$ and $\frac{m}{s^{3}}\frac{1}{\sqrt{Hz}}$ respectively and are characteristics of the IMU. Discretization of the evolution of biases leads to:

(24) $$\begin{array}{cc} {b_{g}}_{k+1} & =-\exp\left(\frac{\Delta t_{ij}}{\tau_{g}}\right){b_{g}}_{k}+\eta_{bg} \end{array}$$

(25) $$\begin{array}{cc} {b_{a}}_{k+1} & =-\exp\left(\frac{\Delta t_{ij}}{\tau_{a}}\right){b_{a}}_{k}+\eta_{ba} \end{array}$$

The $\eta_{x}$ appearing in (24) and (25) will be then modelled as discrete random variables with Gaussian density N(0,W), W being computed as $\left(t_{k+1}-t_{k}\right)W_{c}$ [19] (p. 231).

The presented models discretization exhibits integrals that all have to be computed numerically in order to build the filter on. Normally, the choice of the numerical integration method depends on the required accuracy. In the current implementation though, we take a very simple approach: we assume that the biases, acceleration and magnetic field gradient—the last two are in world frame—are constant between two MIMU sample and use the following identity:

(26) $$\begin{array}{cc} {\tilde{\Delta R}}_{kk+1} & ≃\mathrm{Exp}\left(\left({\tilde{\omega }}_{k}^{b}-{b_{g}}_{k}\right)\Delta t_{k}\right)\;, \end{array}$$

(27) $$\begin{array}{cc} {\tilde{\Delta v}}_{kk+1} & ≃\left({\tilde{a}}_{k}^{b}-{b_{a}}_{k}\right)\Delta t_{k}\;, \end{array}$$

(28) $$\begin{array}{cc} {\tilde{\Delta p}}_{kk+1} & ≃\frac{1}{2}\left({\tilde{a}}_{k}^{b}-{b_{a}}_{k}\right)\Delta t_{k}^{2}\;, \end{array}$$

(29) $$\begin{array}{cc} {\tilde{D}}_{v;kk+1} & ≃\nabla {\tilde{B}}_{k}^{b}\Delta t_{k}\;, \end{array}$$

(30) $$\begin{array}{cc} {\tilde{D}}_{R;kk+1} & ≃\frac{1}{2}\nabla {\tilde{B}}_{k}^{b}\Delta t_{k}^{2}\;, \end{array}$$

(31) $$\begin{array}{cc} {\tilde{M}}_{kk+1} & ≃\nabla {\tilde{B}}_{k}^{b}\frac{1}{2}\left({\tilde{a}}_{k}^{b}-{b_{a}}_{k}\right)\Delta t_{k}^{2}. \end{array}$$

The error induced by this simple integration scheme is limited by the relatively high frequency of MIMU sample (325 Hz).

### 3.4. Sensors Error Model

Sensors errors propagate through the discrete model when used for filtering. We assume that the noisy sensor reading at time *k* (the *input vector* of the filter) can be written:

(32) $${\tilde{u}}_{k}=\underset{\mathrm{measurement}}{\underset{︸}{\left[\begin{array}{c} {\tilde{a}}_{k}^{b}\\ {\tilde{\omega }}_{k}^{b}\\ \mathrm{Vec}\left(\nabla {\tilde{B}}_{k}^{b}\right) \end{array}\right]}}=\underset{\mathrm{real}\;\mathrm{values}}{\underset{︸}{\left[\begin{array}{c} a_{k}\\ \omega_{k}\\ \mathrm{Vec}\left(\nabla B_{k}\right) \end{array}\right]}}+\left[\begin{array}{ccc} I_{3} & 0_{3} & 0_{3\times 5}\\ 0_{3} & I_{3} & 0_{3\times 5}\\ 0_{3} & 0_{3} & P_{\nabla B} \end{array}\right]\underset{\mathrm{input}\;\mathrm{noise}\;\delta u_{k}\in R^{11}}{\underset{︸}{\left[\begin{array}{c} \eta_{a_{k}^{b}}\\ \eta_{\omega_{k}^{b}}\\ \eta_{\nabla B_{k}^{b}} \end{array}\right]}}$$

The noise $\delta u_{k}$ is assumed to be gaussian $\delta u_{k}\propto N\left(0,\Sigma_{u}\right)$ and is a sensor characteristic. We use here:

(33) $$\Sigma_{u}=\left[\begin{array}{ccc} \sigma_{\omega }^{2}I_{3} & 0_{3\times 3} & 0_{3\times 5}\\ 0_{3\times 3} & \sigma_{a}^{2}I_{3} & 0_{3\times 5}\\ 0_{5\times 3} & 0_{5\times 3} & \Sigma_{\nabla B} \end{array}\right]\;\mathrm{with}\;\sigma_{\omega }\;\mathrm{in}\;\frac{rad}{s},\;\sigma_{a}\;\mathrm{in}\;\frac{m}{s^{2}}\;\mathrm{and}\;\Sigma_{\nabla B}\;\mathrm{in}\;\frac{\mathrm{Gauss}}{m}\;\in R^{5\times 5}.$$

Note that $P_{\nabla B}$ reflects that we explicitly exploit the symmetry in the magnetic field from Maxwell equation. They implies that the gradient should be a symmetric matrix and of zero trace and thus has 5 degrees of freedom, instead of the nine coefficient of the full matrix. $P_{\nabla B}\in R^{9\times 5}$ is thus the matrix of the application generating the vectorized gradient matrix from minimal gradient coordinates:

(34) $$\begin{array}{ccc} R^{5} & \to & R^{9}\\ \left(\begin{array}{c} g_{1}\\ g_{2}\\ g_{3}\\ g_{4}\\ g_{5} \end{array}\right) & \mapsto & \mathrm{Vec}\left(\left(\begin{array}{ccc} g_{1} & g_{2} & g_{3}\\ g_{2} & g_{4} & g_{5}\\ g_{3} & g_{5} & -g_{1}-g_{4} \end{array}\right)\right) \end{array}$$

## 4. Tight Fusion Filter

This section describe thoroughly the MI-MSCKF filter that is used in the experimental Section 5. Section 4.1 describes the parametrization of the state of the filter. Section 4.2 is dedicated to the propagation step while Section 4.3 details the magnetic and visual measurement equations.

### 4.1. State and Error State

We define the state space at time index *k*, $X_{k}$ as a manifold which is compound of :

- the *keyframe poses* state space $K_{k}$, which elements are the poses of a set of *N* past frames at time indexes $\left\{i_{1},\ldots i_{N}\right\}$ not necessarily temporally successive but close in time. With poses written $\xi_{i}=\left(R_{i}^{w},p_{i}^{w}\right)$, this part of the state have the following form:
  (35) $$\left(\xi_{i_{1}},\cdots ,\xi_{i_{N}}\right)\in K_{k}={\left(\mathrm{SO}\left(3\right)\times R^{3}\right)}^{N}$$
- the *current mimu state* S space which elements have the following form:
  (36) $$s_{k}={\left(\xi_{k},v_{k}^{b},B_{k}^{b},{b_{a}}_{k},{b_{g}}_{k}\right)}^{T}\in \mathrm{SO}\left(3\right)\times R^{3}\times R^{12}$$

A complete state space element at time index *k* is thus noted:

(37) $$X_{k}=\left(\xi_{i_{1}},\cdots ,\xi_{i_{N}},s_{k}\right)\in X_{k}=K_{k}\times S$$

We use the Lie group structure of this manifold and its tangent space to (i) define the error tracked by the filter and (ii) define the Jacobian of the measurement process. This derives from the fact that a perturbations around an element can be expressed as an element of its Lie algebra. We use the ⊞ operator symbol, so that $X_{k}⊞\delta X_{k}$ computes a new state in $X_{k}$ from a tangent perturbation $\delta X_{k}$ around $X_{k}$. We define it as regular addition operation for all components of the state except for pose states where we use:

(38) $$\xi ⊞\delta \xi =\left(\mathrm{Exp}\left(\delta \xi_{4:6}\right)R,p+\delta \xi_{1:3}\right)$$

Similarly we define the reciprocal operator ⊟ as the binary operator giving the perturbation element between two states of X. It is defined as regular minus operation except for pose states for which it is:

(39) $$\xi_{2}⊟\xi_{1}={\left[\mathrm{Log}{\left(R_{2}R_{1}^{-1}\right)}^{T},p_{2}^{T}-p_{1}^{T}\right]}^{T}$$

We here define the error state as the application ⊟ operator between the true state and the estimated state, noted hereafter with an hat. It is thus an element of the tangent space at the current estimate.

(40) $$\begin{array}{c} \epsilon_{k}\overset{\mathrm{def}}{=}X_{k}⊟{\hat{X}}_{k}\Leftrightarrow X_{k}={\hat{X}}_{k}⊞\epsilon_{k} \end{array}$$

Note that this implies a parametrization of the rotation error in *world* frame, and is different from our previous work [13] where rotation error was parametrized in the body frame.

The filtering process propagates the estimated mean, along with an estimate of uncertainty. This uncertainty is represented as a Gaussian density on the error state $\epsilon_{k}\propto N\left(0,P_{k}\right)$ in order to take advantage of the Lie group structure defined previously, i.e., a minimal parametrization and a locally euclidean structure in tangent space.

For numerical reasons, the covariance $P_{k}$ will be tracked by the filter in an square-root information form, such that we have the relationship $P_{k}={\left({\hat{S}}_{k}^{T}{\hat{S}}_{k}\right)}^{-1}$ with ${\hat{S}}_{k}$ an upper triangular matrix. The next section describes how this quantity evolves through the different steps of the filter: propagation and update.

### 4.2. Propagation/Augmentation/Marginalization

**Figure 3:**
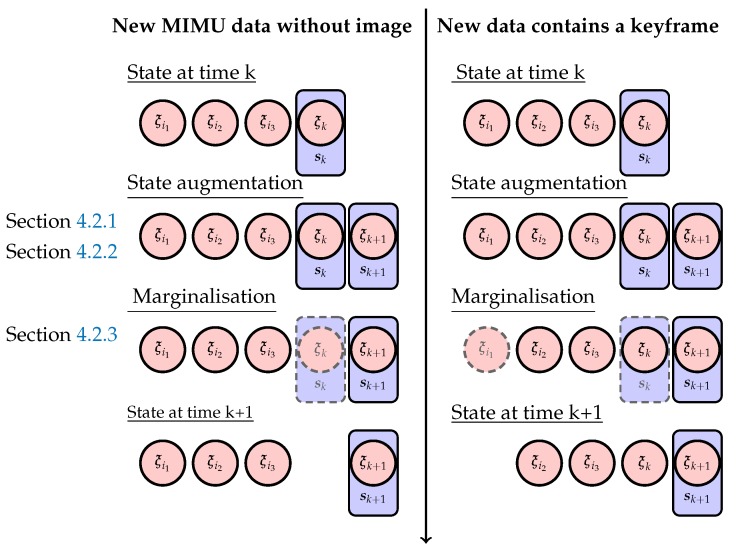
Illustration of state augmentation and marginalization across time as described in Section 4.2.

At the time of arrival of a new IMU data k+1, $X_{k|k}$ and ${\hat{S}}_{k|k}$ are propagated. This is summarized by Figure 3 and splitted in three steps. First, the mimu state $s_{k}$ is propagated with discretized model (Section 4.2.1), then the full state is augmented with the resulting new mimu state $s_{k+1}$ (Section 4.2.2). Finally, some part of this augmented state are marginalized before the update step (Section 4.2.3).

#### 4.2.1. Propagation

Keyframe poses states are estimation of physical quantities blocked at fixed instant in time, their error do not evolve with time and thus they are propagated with an identity function. Besides, the current mimu state error is propagated according to

(41) $$s_{k+1}=f_{\mathrm{mimu}}\left(s_{k},{\tilde{u}}_{k},\eta_{k}\right)$$

where the *discrete mimu process function* $f_{\mathrm{mimu}}$ summarizes (8)–(11) and (24)–(25).

The mimu state error is increased from the three sources of uncertainty: the stochastic model of biases, the measurement noise $\delta u_{k}$ on the input vector and the uncertainty of the previous estimate:

(42) $$\begin{array}{cc} \epsilon_{\mathrm{mimu},k+1} & =f_{\mathrm{mimu}}\left({\hat{s}}_{k}⊞\epsilon_{k}^{\mathrm{mimu}},{\tilde{u}}_{k}-\delta u_{k},\eta_{k}\right)⊟f_{\mathrm{mimu}}\left({\hat{s}}_{k},{\tilde{u}}_{k},0\right) \end{array}$$

(43) $$\begin{array}{c} ≃\Phi_{k+1,k}^{\mathrm{mimu}}\epsilon_{k}^{\mathrm{mimu}}+G_{k+1,k}^{\mathrm{mimu}}\delta u_{k}+C_{k+1,k}^{\mathrm{mimu}}\eta_{k}+O\left(\epsilon_{k}^{\mathrm{mimu}},\delta u_{k},\eta_{k}\right) \end{array}$$

The expression of matrices $\Phi_{k+1,k}^{\mathrm{mimu}}$, $G_{k+1,k}^{\mathrm{mimu}}$, $C_{k+1,k}^{\mathrm{mimu}}$ are derived by 1st order development of (42):

(44) $$\Phi_{k+1,k}^{\mathrm{mimu}}=\left[\begin{array}{cccccc} I_{3} & 0_{3} & 0_{3} & 0_{3} & {\Phi_{k+1,k}^{\mathrm{mimu}}}_{Rb_{g}} & 0_{3}\\ {\left[-R_{k}\left(v_{k}^{b}\Delta t+{\tilde{\Delta p}}_{kk+1}\right)\right]}_{\times } & I_{3} & R_{k}\Delta t & 0_{3} & {\Phi_{k+1,k}^{\mathrm{mimu}}}_{pb_{g}} & {\Phi_{k+1,k}^{\mathrm{mimu}}}_{pb_{g}}\\ R_{k+1}^{T}{\left[g^{w}\right]}_{\times }\Delta t & 0_{3} & \Delta R & 0_{3} & {\Phi_{k+1,k}^{\mathrm{mimu}}}_{vb_{g}} & {\Phi_{k+1,k}^{\mathrm{mimu}}}_{vb_{a}}\\ \Delta R^{T}{\tilde{D}}_{R;kk+1}{\left[g^{w}\right]}_{\times } & 0_{3} & \Delta R^{T}{\tilde{D}}_{v;kk+1} & \Delta R^{T} & {\Phi_{k+1,k}^{\mathrm{mimu}}}_{Bb_{g}} & {\Phi_{k+1,k}^{\mathrm{mimu}}}_{Bb_{a}}\\ 0_{3} & 0_{3} & 0_{3} & 0_{3} & -\exp\left(\frac{\Delta t}{\tau_{g}}\right) & 0_{3}\\ 0_{3} & 0_{3} & 0_{3} & 0_{3} & 0_{3} & -\exp\left(\frac{\Delta t}{\tau_{a}}\right) \end{array}\right]$$

(45) $$\begin{array}{cc} G_{k+1,k}^{\mathrm{mimu}}=\left[\begin{array}{ccc} -{\Phi_{k+1,k}^{\mathrm{mimu}}}_{Rb_{g}} & 0 & 0\\ -{\Phi_{k+1,k}^{\mathrm{mimu}}}_{pb_{g}} & -{\Phi_{k+1,k}^{\mathrm{mimu}}}_{pb_{a}} & 0\\ -{\Phi_{k+1,k}^{\mathrm{mimu}}}_{vb_{g}} & -{\Phi_{k+1,k}^{\mathrm{mimu}}}_{vb_{a}} & 0\\ -{\Phi_{k+1,k}^{\mathrm{mimu}}}_{Bb_{g}} & -{\Phi_{k+1,k}^{\mathrm{mimu}}}_{Bb_{a}} & {G_{k+1,k}^{\mathrm{mimu}}}_{B\nabla B}\\ 0 & 0 & 0\\ 0 & 0 & 0 \end{array}\right] & C_{k+1,k}^{\mathrm{mimu}}=\left[\begin{array}{cc} 0 & 0\\ 0 & 0\\ 0 & 0\\ 0 & 0\\ I_{3} & 0\\ 0 & I_{3} \end{array}\right] \end{array}$$

(46) $$\begin{array}{cc} {\Phi_{k+1,k}^{\mathrm{mimu}}}_{Rb_{g}} & =-R_{k}\Delta t \end{array}$$

(47) $$\begin{array}{cc} {\Phi_{k+1,k}^{\mathrm{mimu}}}_{vb_{g}} & =-\Delta t{\left[v_{k+1}\right]}_{\times }+\partial_{{b_{g}}_{k}}\left({\tilde{\Delta v}}_{kk+1}\right) \end{array}$$

(48) $$\begin{array}{cc} {\Phi_{k+1,k}^{\mathrm{mimu}}}_{Bb_{g}}= & -\Delta t{\left[B_{k+1}\right]}_{\times }\\ & +\Delta R^{T}\left(v_{k}^{T}⊗I_{3}\right)\partial_{b_{g}}\left(\mathrm{Vec}\left({\tilde{D}}_{v;kk+1}\right)\right)\\ & +\Delta R^{T}\left(g^{T}R_{k}⊗I_{3}\right)\partial_{b_{g}}\left(\mathrm{Vec}\left({\tilde{D}}_{R;kk+1}\right)\right) \end{array}$$

(49) $$\begin{array}{c} +\Delta R^{T}\partial_{b_{g}}\left({\tilde{M}}_{kk+1}\right) \end{array}$$

(50) $$\begin{array}{cc} {\Phi_{k+1,k}^{\mathrm{mimu}}}_{pb_{g}} & =R_{k}\partial_{{b_{g}}_{k}}\left({\tilde{\Delta p}}_{kk+1}\right) \end{array}$$

(51) $$\begin{array}{cc} {\Phi_{k+1,k}^{\mathrm{mimu}}}_{vb_{a}} & =\Delta R^{T}\partial_{{b_{a}}_{k}}\left({\tilde{\Delta v}}_{kk+1}\right) \end{array}$$

(52) $$\begin{array}{cc} {\Phi_{k+1,k}^{\mathrm{mimu}}}_{pb_{a}} & =R_{k}\partial_{{b_{a}}_{k}}\left({\tilde{\Delta p}}_{kk+1}\right) \end{array}$$

(53) $$\begin{array}{cc} {\Phi_{k+1,k}^{\mathrm{mimu}}}_{Bb_{a}} & =\Delta R^{T}\partial_{{b_{a}}_{k}}\left({\tilde{M}}_{kk+1}\right)\\ {G_{k+1,k}^{\mathrm{mimu}}}_{B\nabla B} & =\Delta R^{T}\left(v_{k}^{T}⊗I_{3}\right)\partial_{\nabla B}\left(\mathrm{Vec}\left({\tilde{D}}_{v;kk+1}\right)\right) \end{array}$$

(54) $$\begin{array}{c} \;+\Delta R^{T}\left(g^{T}R_{k}⊗I_{3}\right)\partial_{\nabla B}\left(\mathrm{Vec}\left({\tilde{D}}_{R;kk+1}\right)\right)P_{\nabla B}+\Delta R^{T}\partial_{\nabla B}\left({\tilde{M}}_{kk+1}\right)P_{\nabla B} \end{array}$$

where $P_{\nabla B}$ is defined as in (34). Note that we wrote here the transition and noise matrices as a function of the integrals (12)–(17), independently of the way the are computed, so that these expressions are still valid if one choose to compute the integrals with a more sophisticated scheme. Derivative of these integrals with respect to the biases are also required: these quantities should be computed simultaneously with the integrals. In our implementation, they are computed easily with the approximations made in (26)–(31).

#### 4.2.2. State Augmentation

When MIMU sample k+1 arrives, the mimu propagation function is used to augment the state with the new current mimu state ${\hat{s}}_{k+1}=f_{\mathrm{mimu}}\left({\hat{s}}_{k},{\tilde{u}}_{k},0\right)$, leading to the augmented state ${\hat{X}}_{k+1|k}^{⊕}$. The error state square root information matrix is augmented accordingly to [14], ${\hat{S}}_{k+1|k}^{⊕}$ using the Jacobian derived in previous subsection.

(55) $$\begin{array}{c} {\hat{X}}_{k+1|k}^{⊕}\overset{\mathrm{def}}{=}\left(X_{k|k},s_{k+1}\right) \end{array}$$

(56) $$\begin{array}{c} {\hat{S}}_{k+1|k}^{⊕}\overset{\mathrm{def}}{=}\left[\begin{array}{cc} {\hat{S}}_{k|k} & 0\\ V_{1} & V_{2} \end{array}\right] \end{array}$$

with

(57) $$\begin{array}{cc} V_{1} & =[0_{18\times 6},\cdots ,0_{18\times 6},Q_{k+1}^{-\frac{1}{2}}{\Phi_{k+1,k}^{\mathrm{mimu}}}_{k+1,k}] \end{array}$$

(58) $$\begin{array}{cc} V_{2} & =Q_{k+1}^{-\frac{1}{2}} \end{array}$$

and the discrete model noise at $Q_{k}$ time *k* :

(59) $$Q_{k+1}=\left(\begin{array}{c} G_{k+1,k}^{\mathrm{mimu}}C_{k+1,k}^{\mathrm{mimu}} \end{array}\right)\left(\begin{array}{cc} \Sigma_{u} & 0\\ 0 & W \end{array}\right)\left(\begin{array}{c} {G_{k+1,k}^{\mathrm{mimu}}}^{T}\\ {C_{k+1,k}^{\mathrm{mimu}}}^{T} \end{array}\right).$$

#### 4.2.3. Marginalization of Old State

Then, in order to bound the size of ${\hat{X}}_{k+1|k}$, some part of it are marginalized. The state elements to be marginalized depend on the type of data available at the current timestamps as depicted in Figure 3. If only MIMU data (without an new keyframe image attached) are arriving at time *k*, $s_{k}$ is marginalized. If MIMU data and a new keyframe image are available at time *k*, the oldest keyframe pose is marginalized together with the non pose element of $s_{k}$. Note that since the image frame rate is well below MIMU frequency, the first case happens more often than the second case.

Within the square root information form, marginalization is done similarly to [14]. With $\Pi_{k}$ being the matrix permutation putting the *to marginalize* error states at the beginning, a QR decomposition of a square root information matrix of the permuted augmented error state vector writes

(60) $$\begin{array}{cc} {\hat{S}}_{k+1|k}^{⊕}\Pi_{k} & =O_{p}R_{p},\;\begin{array}{c} O_{p}\in O\left(n\right)\\ R_{p}\in R^{n} \end{array} \end{array}$$

(61) $$\begin{array}{c} =O_{p}\left[\begin{array}{cc} {}* & *\\ 0 & {\hat{S}}_{k+1|k} \end{array}\right] \end{array}$$

We obtain the predicted state ${\hat{X}}_{k+1|k}$ removing marginalized states, and its—upper triangular—square-root information matrix ${\hat{S}}_{k+1|k}$. If no measurement update has to be performed at current time step they can be used directly as $X_{k+1|k+1}$ and ${\hat{S}}_{k+1|k+1}$ for the next propagation.

Note: The marginalization of a joint Gaussian distribution with a square-root information form is not often demonstrated in book or lecture but can be deduced from the full information form $\Lambda_{\mathrm{joint}}$:

(62) $$\begin{array}{cccc} \mathrm{if}\;\Lambda_{\mathrm{joint}} & =\left[\begin{array}{cc} \Lambda_{M} & \Lambda_{MR}\\ \Lambda_{MR}^{T} & \Lambda_{R} \end{array}\right]={\hat{S}}_{\mathrm{joint}}^{T}{\hat{S}}_{\mathrm{joint}}=\left[\begin{array}{cc} {\hat{S}}_{M}^{T}{\hat{S}}_{M} & {\hat{S}}_{M}^{T}{\hat{S}}_{MR}\\ {\hat{S}}_{MR}^{T}{\hat{S}}_{M} & {\hat{S}}_{R}^{T}{\hat{S}}_{R}+{\hat{S}}_{MR}^{T}{\hat{S}}_{MR} \end{array}\right] & \mathrm{with}\;{\hat{S}}_{\mathrm{joint}} & =\left[\begin{array}{cc} {\hat{S}}_{M} & {\hat{S}}_{MR}\\ 0 & {\hat{S}}_{R} \end{array}\right] \end{array}$$

then the square root information matrix resulting from marginalization of the the *M* variables is: ${\hat{S}}_{R}^{\mathrm{marg}}={\hat{S}}_{R.}$ This is deduced by calculus from the usually demonstrated fact that: $\Lambda_{R}^{\mathrm{marg}}=\Lambda_{R}-\Lambda_{MR}\Lambda_{M}^{-1}\Lambda_{RM.}^{T}$

### 4.3. Measurement Update

The filter processes two kinds of measurement: (i) the magnetic one that compares the magnetic field measured at the current timestamp with the magnetic field predicted by the filter; (ii) the visual measurement equation for features for which tracking has just ended.

We first briefly recall the update process of the inverse square root filter on a manifold. Let us suppose that some measurement occurs that can be modeled as:

(63) $$\begin{array}{c} h\left(X_{k+1}\right)={\tilde{h}}_{k+1}+\eta_{h}\in R^{n_{m}},\;\eta_{h}∼N\left(0,\Sigma_{h}\right) \end{array}$$

with $n_{m}$ the dimension of the measurement vector. Writing the dimension of the predicted state as $n_{s}$, the measurement error $z_{k+1}=h\left({\hat{X}}_{k+1|k}\right)-{\tilde{h}}_{k+1}$ and H the jacobian of the application:

(64) $$\begin{array}{c} \begin{array}{ccc} R^{n_{s}} & \to & R^{n_{m}}\\ \delta X & \mapsto & h({\hat{X}}_{k+1|k}⊞\delta X) \end{array}, \end{array}$$

the update step finds the tangent correction $\delta X^{*}$ that minimizes the following linearized cost:

(65) $$\begin{array}{cc} C(\delta X) & ={∥\delta X∥}_{P_{k+1|k}}^{2}+{∥H\delta X-z_{k+1}∥}_{\Sigma_{h}}^{2}\\ & ={∥{\hat{S}}_{k+1|k}.\delta X∥}^{2}+{∥\Sigma_{h}^{-\frac{1}{2}}\left(H\delta X-z_{k+1}\right)∥}^{2}\\ & ={∥\left[\begin{array}{c} {\hat{S}}_{k+1|k}\\ \Sigma_{h}^{-\frac{1}{2}}H \end{array}\right]\delta X-\left[\begin{array}{c} 0\\ \Sigma_{h}^{-\frac{1}{2}}z_{k+1} \end{array}\right]∥}^{2}. \end{array}$$

The optimum point can be obtained by of a thin QR decomposition:

(66) $$\left[\begin{array}{c} {\hat{S}}_{k+1|k}\\ \Sigma_{h}^{-\frac{1}{2}}H \end{array}\right]=O_{u}R_{u},\;\begin{array}{c} O_{u}\in O\left(n_{s}+n_{m}\right)\\ R_{u}\in R^{n_{s}+n_{m}} \end{array}$$

(67) $$C\left(\delta X\right)={∥R_{u}\delta X-O_{u}^{T}\left[\begin{array}{c} 0\\ \Sigma_{h}^{-\frac{1}{2}}z_{k+1} \end{array}\right]∥}^{2},$$

$R_{u}$ being upper triangular, $\delta X^{*}$ is efficiently computed by back-substitution. This optimal correction is finally applied to the predicted state with the retraction operator :

(68) $$\begin{array}{cc} X_{k+1|k+1} & ={\hat{X}}_{k+1|k}⊞\delta X^{*} \end{array}$$

(69) $$\begin{array}{cc} {\hat{S}}_{k+1|k} & =R_{u}J_{r}^{-1}. \end{array}$$

With $J_{r}$ being the Jacobian at *e* = 0 of:

(70) $$\begin{array}{cc} R^{n_{s}} & \to R^{n_{s}}\\ e & \mapsto \left({\hat{X}}_{k+1|k}⊞\left(\delta X^{*}+e\right)\right)⊟\left({\hat{X}}_{k+1|k}⊞\delta X^{*}\right). \end{array}$$

Intuitively, this Jacobian transforms the square-root information matrix from the tangent space of predicted state to the tangent space of the updated state. Note that, in the current parametrization choice (cf. (38)), this Jacobian is the identity matrix; this was not the case in our previous work [13] where the rotation error was expressed in body frame.

#### 4.3.1. Magnetic Measurement Update

The magnetic measurement update is the simplest and uses at MIMU frequency the direct magnetic field measurement which writes:

(71) $$\begin{array}{cc} h_{B}\left(X_{k}\right) & =P_{B_{b}}X_{k} \end{array}$$

(72) $$\begin{array}{cc} \Sigma_{h_{B}} & =\sigma_{B}^{2}I_{3} \end{array}$$

with $P_{B_{b}}$ the projection operator from the state space to the coordinates of the state corresponding to $B_{b}$. $\sigma_{B}$ is the noise of the magnetometers reading.

#### 4.3.2. Opportunistic Feature Tracks Measurement Update

We use the feature tracks in the same way as proposed by [20]. We derive here the equation for completeness.

When a feature track ends or when the frame in which the feature was detected is about to be marginalized, we process the entire feature track as a measurement constraining the pose of each in-state frame where the feature was detected.

The predicted reprojection of feature *i* in frame at time *t* is written:

(73) $$\begin{array}{c} {\mathrm{pr}}_{t}^{i}\left(\xi_{t},l_{i}^{w}\right)=\pi \left(R_{c}^{b}T\left({R_{t}^{w}}^{T}\left(l_{i}^{w}-p_{t}^{w}\right)-p_{c}^{b}\right)\right)\in R^{2} \end{array}$$

with $l_{i}^{w}\in R^{3}$ the 3D-position of the features *i* in inertial coordinates and $\pi :R^{3}\;\to \;R^{2}$ the projection function of the camera. We recall that $\left(R_{c}^{b},p_{c}^{b}\right)$ is the known transform between the body frame and the camera frame. $l_{i}^{w}$ is computed by a fast triangulation function from known in-state poses and the measured observation, noted $o_{it}$.

By stacking all these reprojections, we can write the non linear measurement function:

(74) $$\begin{array}{cc} h_{fi}\left(X,l_{i}^{w}\right) & =\left[\begin{array}{c} \cdot \\ {\mathrm{pr}}_{t}^{i}\left(\xi_{t},l_{i}^{w}\right)\\ \cdot \end{array}\right]=\left[\begin{array}{c} \cdot \\ o_{it}\\ \cdot \end{array}\right]+\eta_{fi}\;, \end{array}$$

with $\eta_{fi}$ is assumed to be an additive Gaussian white noise : $\eta_{fi}∼N\left(0,\Sigma_{C}\right)$, $\Sigma_{C}=\sigma_{c}I_{2m}$. We note subsequently $o_{i}$ the vector resulting from stacking of 2-vector observation $o_{it}$.

The attentive reader may note that this measurement equation is not of the form of (63), because $l_{i}^{w}$ is not part of our state: for this reason we can not directly use (65) and (67). Worse, the computed $l^{w}$ estimate is correlated with the current state error, so that we can not just use it as a fixed constant. The solution used here aims at expressing a projection of the residual that depends only on the poses, up to the first order.

We start by linearizing the residual then proceeds the minimization in two steps to extract the used measurement equation. Linearization of $r_{i}:X,l_{i}^{w}\mapsto \left(h_{fi}\left(X,l_{i}^{w}\right)-o_{i}\right)$ yields:

(75) $$\begin{array}{c} r_{i}\left(X_{k+1|k}⊞\delta X,l_{i}^{w}+\delta l_{i}^{w}\right)\\ \;≃F_{i}\delta X+E_{f}\delta l_{i}^{w}+\left(h_{fi}\left(X_{k+1|k},l_{i}^{w}\right)-o_{i}\right)\\ \;=F_{k}\delta X+E_{f}\delta l_{i}^{w}-\delta o_{i} \end{array}$$

$\delta o_{i}=h_{fi}\left(X_{k+1|k},l_{i}^{w}\right)-o_{i}$ is the 2m-vector of predicted residual error. $E_{f}$ is a 2m×3 matrix of rank 3 ; *m* denoting the number of observations for the feature. The rank of $E_{f}$ is guaranteed during the triangulation. Its QR decomposition writes:

(76) $$\begin{array}{cc} E_{f} & =\left[O_{E1},O_{E0}\right]\left[\begin{array}{c} R_{E1}\\ 0_{2m-3\times 2m} \end{array}\right] \end{array}$$

(77) $$\begin{array}{cc} O_{E1}\in R^{2m\times 3},\; & O_{E0}\in R^{2m\times 2m-3},R_{E1}\in GL_{3}\left(R\right); \end{array}$$

Properties of square orthogonal matrices on the $L_{2}$ norm of vectors allow to split the cost function into two terms, one depending only of the current predicted state vector.

(78) $$\begin{array}{c} \min_{\delta X,\delta l_{i}^{w}}{∥r_{i}{∥}^{2}}_{\Sigma_{C}}=\min_{\delta X,\delta l_{i}^{w}}{{∥\left[\begin{array}{c} O_{E1}^{T}\\ O_{E0}^{T} \end{array}\right]r_{i}∥}^{2}}_{\Sigma_{C}}\\ =\min_{\delta X,\delta l_{i}^{w}}{∥O_{E1}^{T}F_{i}\delta X+R_{E1}\delta l_{i}^{w}-O_{E1}^{T}\delta o_{i}∥}_{\Sigma_{C}}^{2}\\ +\min_{\delta X}{∥Q_{E0}^{T}F_{i}\delta X-O_{E0}^{T}\delta o_{i}∥}_{\Sigma_{C}}^{2} \end{array}$$

As $R_{E1}$ is invertible, the first term of the quantity to be minimized can be reduced to zero for all δX. Minimization reduces thus to:

(79) $$\begin{array}{c} \min_{\delta X}{∥O_{E0}^{T}F_{i}\delta X-O_{E0}^{T}\delta o_{i}∥}_{\Sigma_{C}}^{2} \end{array}$$

Finally, the previous linearized residual is used in the cost function (65), i.e., we use for the H matrix, the z error vector and the covariance of measurement $\Sigma_{h}$ the quantities:

(80) $$\begin{array}{cccccc} H_{f} & =O_{E0}^{T}F_{i}, & z_{f} & =O_{E0}^{T}\delta o_{i}, & \Sigma_{h_{f}} & =\sigma_{C}I_{2m-3}. \end{array}$$

Note that everything happens here as if we introduced the features position into the state, but instantly marginalized it.

### 4.4. Filter Initialization

One sensitive issue in a VINS filter is initialization. It usually involves some specific algorithm as described in [21,22] for instance. In our implementation, we proceed as follows: after switch on, the current state is initialized at origin with an attitude matching the current acceleration direction and a zero speed, both with high variance. The filter is then ran using the high frequency magnetic update equation in order to get rapidly a stabilized trajectory from the first few seconds. This trajectory is used to bootstrap the first keyframe poses state and then to start using features information. This initialization process relies on the empirical observation that the MI-DR filter convergence basin is large. Admittedly, it would degrade severely the filter if the system’s switch on occurs in an area where magneto-inertial dead-reckoning is not reliable.

## 5. Experimental Study

### 5.1. Hardware Prototype Description and Data Syncing

**Figure 4:**
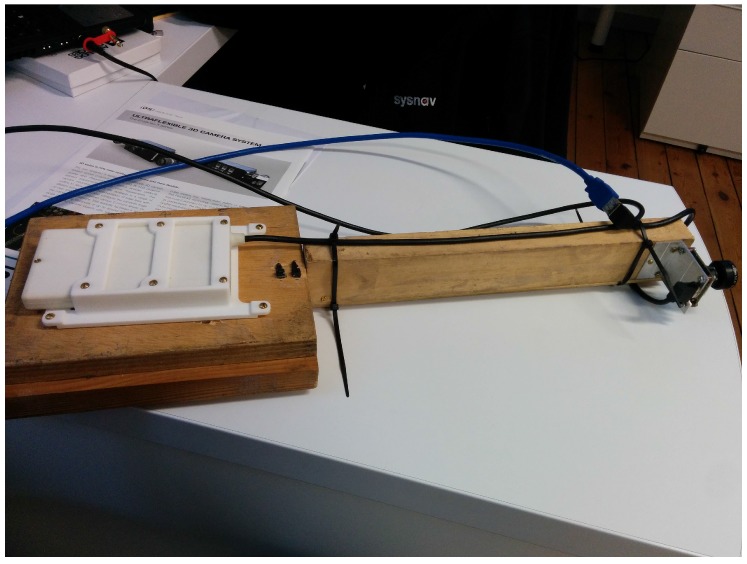
The sensor setup used in this work. The white box on the left side contains the MIMU sensor, the camera is on the right side. Both sensors are rigidly attached through a non-magnetic, non conductive material (wood).

The sensor system is pictured on Figure 4. The camera is rigidly attached 47 cm away of the MIMU system to avoid any potential magnetic perturbation. Such a large distance is necessary because the off-the-shelf camera has not been specifically designed for reducing its magnetic footprint. Reducing the hardware to a wearable size would involve sensors co-design that was not in the scope of this work. For the vision part, we use an IDS uEye 3241-LE equipped with a Lensagon BM4018S118 lens. It provides around 100 degrees of field of view. Camera intrinsics and extrinsic parameters are calibrated with the Kalibr toolbox [23]. The MIMU provides data at 325 Hz, the camera at 20 Hz. Magnetometers, accelerometers and gyro-meters are all MEMS sensors digitized with sigma-delta ADCs which were carefully calibrated.

The camera and MIMU provide timestamps computed from different clocks. We synchronize them offline: timestamps shifts are estimated both at start and at the end of the records by the checkerboard-based Kalibr calibration toolbox; clock drift is then deduced and corrected for.

The camera exposure time is fixed at 10 milliseconds maximum, reducing worst case motion blur and allowing to timestamp accordingly each image observation. However, this choice limits the camera’s ability to adapt to a very low-light environment.

### 5.2. Filter Parameters Tuning

Most parameters of the filter are chosen in a deterministic and consistent fashion. MIMU noise standard deviation $\sigma_{a},\sigma_{\omega },\sigma_{B}$ and biases evolution parameters $\tau_{bg},\tau_{ba},\sigma_{bg;c},\sigma_{ba;c}$ are derived from sensors characteristics measured empirically with an Allan standard deviation. The pixel reprojection noise $\sigma_{C}$ is set to $\sqrt{2}$, which is the diagonal size of a pixel. Only the magnetic part of covariance $\Sigma_{\nabla B}$ is tuned empirically. It is set greater than what would derive from the covariance of magnetometers white noise, so as to absorb some modeling errors of the magnetic field, such as small non-stationarities or high values of the 2nd order spatial term. Note that parameter tuning is unchanged for all presented datasets in order to draw fair conclusions.

### 5.3. Visual Processing Implementation

The visual processing pipeline aims at constantly track 200 interest points well spread in the image. In order to enforce a good repartition of corners across the entire image, we use a bucket strategy. Harris-corner response is computed over the entire image and we retain only the strongest features in buckets for which the number of already tracked points is below a threshold. We use here a partition of the image in 6×8 buckets. Detected corners are tracked from frame to frame with OpenCV pyramidal KLT algorithm until either:

- they go out of the field of view;
- the tracking fails;
- they are classified as outliers;
- the frame where they were firstly detected is to be marginalized at next propagation step.

We ran a 2-point RANSAC algorithm between subsequent frames for outliers detection and rejection, using the relative orientation from the integrated gyroscope as rotation between the two frames.

An ended feature track is used as measurement, only if:

- it spans at least three poses;
- its initial triangulation did not exhibit any degeneracy;
- its re-projection error is below a threshold.

Contrarily to our previous implementation [13], this threshold is dynamically set with a $\chi^{2}$ threshold. As a result, the criterion becomes looser when the estimated uncertainty of relative poses increases.

**Figure 5:**
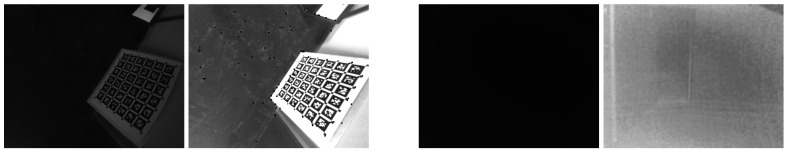
Image processing pipeline. (**Left**): Raw input images. (**Right**): after rectification, intensity normalization, and corner detection. On the second example, the normalization reveals a faint signal, but it is too noisy for corner detection to works.

In order to make the visual pipeline more robust to dark areas, we normalize each input image by its averaged intensity before corner detection and tracking. Some very dark images then become usable for corner detection despite a significant increase of the photometric noise which affect them. Noise amplification leads to spurious features track, but these are most often correctly rejected by our outlier rejection strategy. Overall, we found that normalization significantly improves the performance of pure MSCKF VINS algorithm on our dataset. Some raw/normalized images are presented in Figure 5.

Note that, in contrast with the tuning of the filter, the parameters of the vision pipeline (KLT window size, number of octaves in the pyramid of KLT, RANSAC threshold and minimum Harris score for corner detection) were chosen empirically.

### 5.4. Trajectory Evaluation

#### 5.4.1. Dataset Presentation

**Figure 6:**
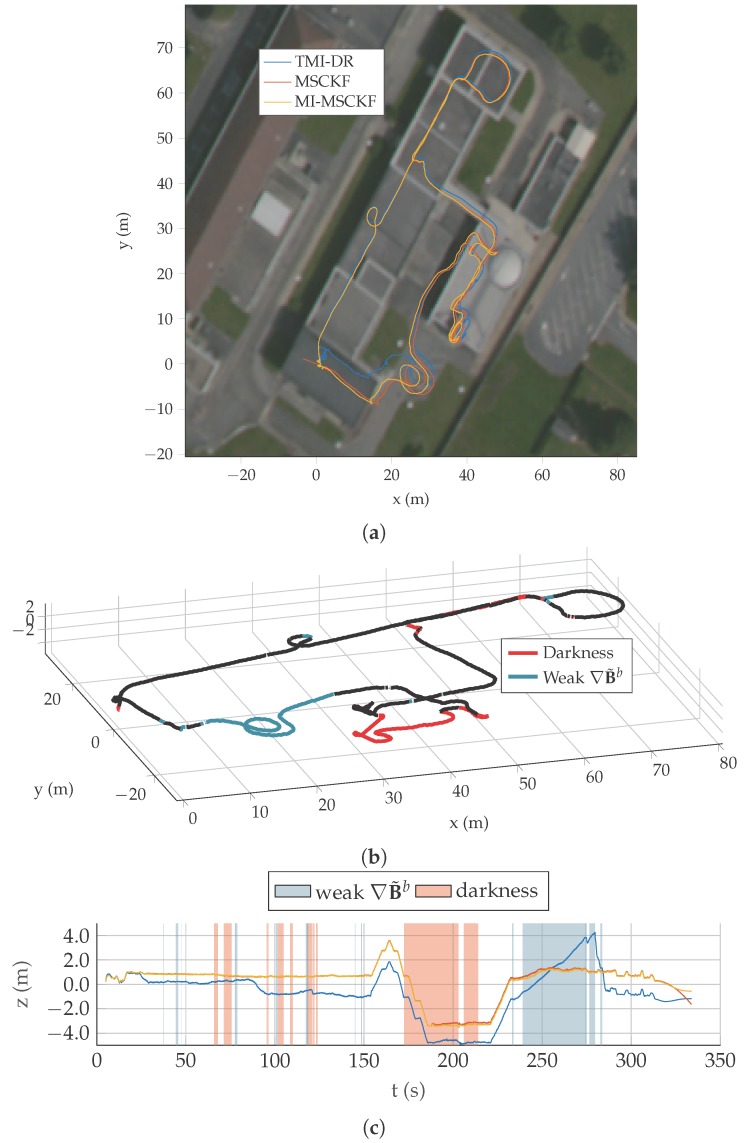
(**a**) Overview of trajectory Traj2 as reconstructed by the three filters. (**b**) Visualisation of dark areas and low-gradient areas over the entire trajectory surimposed on MI-MSCKF estimate. (**c**) Height profile of the three estimators on this trajectory.

**Figure 7:**
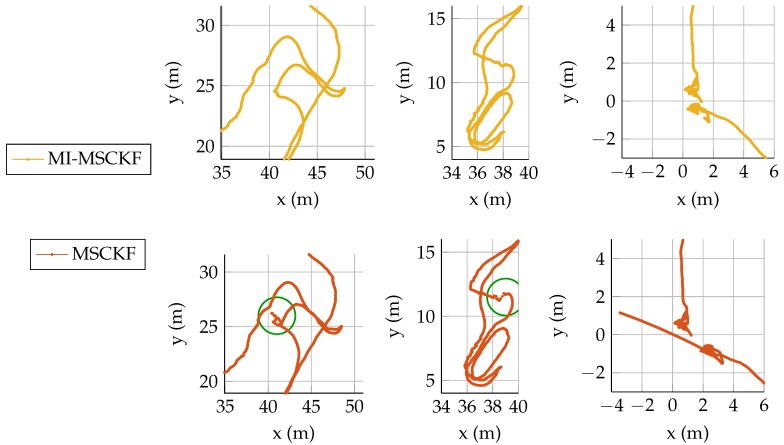
Details of estimation results on Traj2 showing a different behavior between the MSCKF and MI-MSCKF filter. **Left and middle plots**: while transitioning from dark area to lit environment some strong filter correction happen for the MSCKF and lead to discontinuities of the position estimate. In the same areas, MI-MSCKF stays smoother. **Right plot**: here the device is laid on the ground at the end of the trajectory. A large drift of MSCKF occurs, as visual information does not provide any feedback on position. Here again, the MI-MSCKF appears more stable.

**Figure 8:**
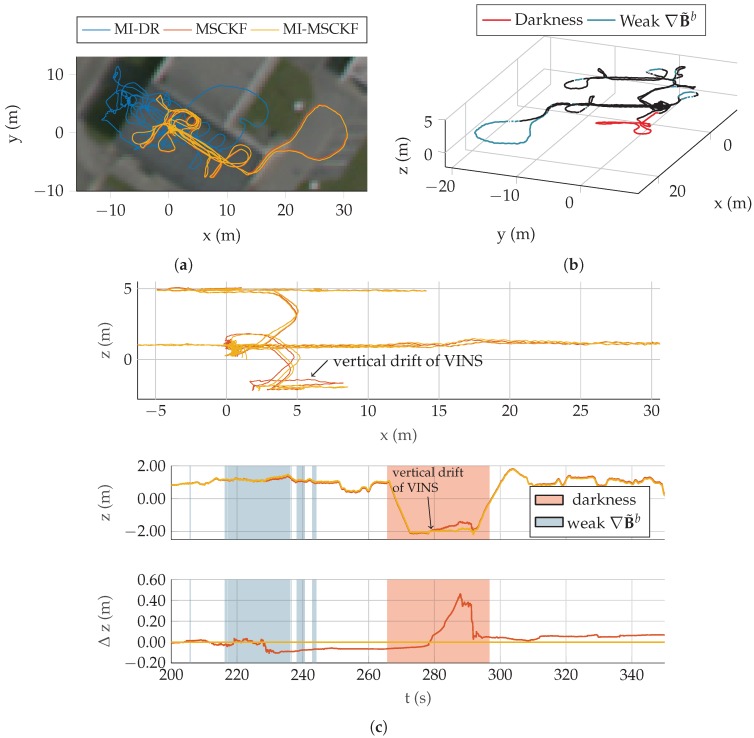
(**a**) Overview of trajectory Traj2 as reconstructed by the three filters. (**b**) Visualisation of dark areas and low-gradient areas over the entire trajectory surimposed on MI-MSCKF estimate. (**c**) Height profile of the three estimators on this trajectory.

**Figure 9:**
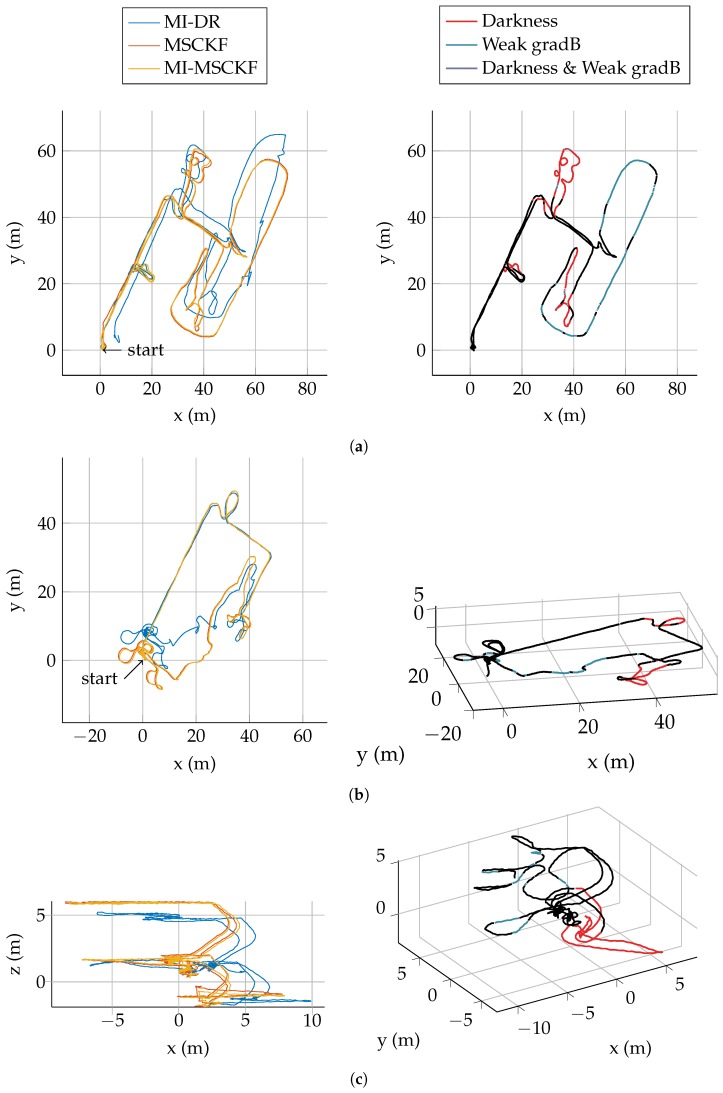
Summary of trajectories on the remaining sequences of the dataset. (**Left**): Estimate of the three configuration of our filter. (**Right**) Color coded MI-MSCKF trajectory showing areas of weak gradient and weak illumination. (**a**) **Traj1** Length: ∼530 m; (**b**) **Traj3** Length: ∼368 m; (**c**) **Traj4** Length: ∼180 m.

We evaluate our algorithm on a dataset of five test trajectories. A pedestrian is carrying the system depicted in Figure 4 and walks through an industrial facility. Trajectories are specifically designed to be challenging for MI-DR and VINS: they are partly done outdoor, with very low magnetic field gradient and they also contains portions visually non informative made in the non lit basement of the building. Detailed results on Traj2 and Traj5 are presented on Figure 6, Figure 7 and Figure 8, the others being more briefly depicted in Figure 9. In all cases, a dedicated plot indicates with a color code the parts of the trajectory where the magnetic field gradient vanishes and parts of the trajectories where the mean intensity is low.

#### 5.4.2. Overall Comparison

Three estimators derived from the presented filter have been compared. The MI-DR is the presented filter without any visual update. MSCKF is the presented filter without any magnetic update. MI-MSCKF is the proposed filter fusing both information. Moreover, we also compare our results with the state of the art VINS filter of [24].

Unfortunately, we do not have access to a ground truth trajectory for our datasets. We then consider three evaluation criteria:

- the estimated trajectories are superimposed to a georeferenced orthoimage in which one pixel represents precisely 0.5 m. We compute an alignment of the trajectory when the checkerboard detected for the first time in the sequence. This alignement results from setting manually the *position and heading* of the checkerboard frame relative to the coordinates system of the satellite image. Note that no manual scale alignment has been made, hence this visualization allows to evaluate roughly the correctness of the global scale of the estimate, for instance on Figure 6a;
- the z profile is globally known as the pedestrian walks along flat corridors—except when he takes stairs to change levels;
- a translational error is computed each time the system comes back to its initial position, thanks to a static checkerboard placed at starting point. This criterion can be visualized in Figure 6c for Traj2 where it is clear the MI-DR estimate is less stable vertically over the entire trajectory.

The last criterion can be quantitavely evaluated: results are displayed in Table 1 with an error given in percentage of the trajectory length. Next sections emphasize some differences in the behaviors of the tested methods.

#### 5.4.3. The Fused Estimate Improves MI-DR in Outdoor Trajectories

The Figure 6 shows the three versions of our filter on Traj2. The trajectory estimated by MI-DR is very close to the others until some point in the outdoor part—note that the outdoor part corresponds to the weak gradient part of the trajectory as depicted on Figure 6b. During this outdoor part, as expected, the MI-DR drifts away compared to the two vision-based estimates which directly leads to a higher final translation error. The same effect is also clearly visible on Traj3, see Figure 9b.

#### 5.4.4. Data Fusion Improves Local Consistency

By reducing the drift in dark areas or low gradient areas, the fused estimates improve the local position estimate consistency, an effect which is not always visible on the metrics of Table 1.

The benefit of magnetometry information in this sense is demonstrated in the details of results on the Traj2 displayed in Figure 7. The two left plots show a similar situation: the VINS estimate (red) is for a few seconds strongly corrected by the filter, leading to non continuous estimates (see green circles). The MI-MSCKF filter stays smoother during the entire trajectories, thanks to the speed observability provided by Equation (7). Interestingly, the pure VINS estimate joins the MI-MSCKF estimate later in the sequence, which makes the temporary drift mostly invisible in the final loop error metric of Table 1. This correction happens when visual information becomes available again. It means that the VINS filter is still able to correct itself after reasonable drift through the information stored in the prior.

The same effect occurs on all trajectories. Consider for instance the trajectory Traj5 depicted on Figure 8, where the lowest part goes also through the dark basement of the building. The MSCKF drifts vertically before being corrected when the pedestrian takes the stair up again. This effect is depicted on details in Figure 8c, which clarifies the evolution of the estimated height with respect to time.

In turn, visual information also helps trajectory consistency. It is clearly visible on the strong vertical drift of MI-DR displayd on Figure 6c around 250 s. Again, this drift is corrected as soon as magnetic gradients becomes sufficiently high to make speed observable again. Note the horizontal drift was never corrected though, leading to the larger final drift shown in Table 1.

The reader may have noticed at that point that Figure 8c shows that MI-DR fails badly on Traj5. Yet, we would like to stress that the fully fused estimate is still able to outperform the VINS estimate, leveraging magnetic information correctly beyond the breaking point of MI-DR. The reason of the failure of MI-DR is an unstationary local magnetic field in the first few seconds of Traj5. It perturbes the MI-DR initialization, which has dramatic consequence on all the rest of the trajectory.

The fact that the fused estimate prevents local drift could be, in our opinion, highly beneficial to the long-term performance in a Extended Kalman Filter strategy. Indeed, it could reduce overall linearization errors and the maximum magnitude of corrections, which are recognized, in VINS community, as an important drawback of filtering approaches compared to bundle adjustment or optimization-based methods.

#### 5.4.5. Comparison with a State of the Art Filter

We also ran the released binary version (Available on https://github.com/MARSLab-UMN/MARS-VINS, we used the commit 8531daf.) of [24] on our dataset. Indeed, we think it is the state of the art in VINS filters for pedestrian navigation. As [24] takes only stereo images (even if their method works well with monocular setup also, as said in the paper) we had to trick slightly their software to use a monocular input. Note that, to be as fair as possible, we have entered in their code the same normalized monocular images we use. In doing so, we observed that normalization has also drastically improved the performance of their filter on our data.

An in-depth and comprehensive comparison between the two implementation is difficult as the code of [24] is not open. If inertial handling in both implementation should be close, the visual pipeline is very sensitive to parameters value and implementation details that are not known by us. Nevertheless, Table 1 demonstrates that both filters compare reasonably and are clearly below 1% of trajectory length error.

We also observed in [24] implementation the presence of strong filters correction after dark areas, as in described in Section 5.4.4. It indicates that this local consistency problem, which is essentially solved by the proposed fused estimate, is indeed a general issue of all VINS filters.

## 6. Conclusions

This work presented a filter to fuse information from a magnetometer array with other sensors traditionally used in VINS. We described in detail, the method we used and discussed its results on real datasets. Comparing the results of three estimators—one using only magnetic-inertial information, one using only visual-inertial information and one fusing both informations—we showed that the fused estimate leads to a more robust trajectory estimate. First, our fused estimate is able to reconstruct the trajectory outdoor where MI-DR techniques breaks because of the lack of gradient; secondly, our system avoids the unrealistic trajectory correction of VINS after significant duration without a good illumination. One trajectory also highlighted the need for either robust estimation technique or outlier rejection scheme for magnetic information. This should deserve more work in the future.

We think that the proposed approach could help to improve localization systems for augmented reality (AR) that are currently using VINS with consumer grade cameras and IMUs. Not only this combination extends the applicability domain of traditional VINS to degraded environments, but we also foresee opportunities to reduce power consumption. Indeed, taking advantages of the good trajectories given by the MI-DR in various conditions, it might be possible to reduce the computational load of the visual pipeline, which could be of major interest in practical applications.

## Figures and Tables

**Table 1:** Summary of final drift error on full dataset (% of trajectory length).

	Traj1	Traj2	Traj3	Traj4	Traj5
MI-DR	1.11	1.98	1.81	1.54	2.87
MSCKF (VINS)	0.33	0.63	0.59	1.05	0.21
MI-MSCKF	**0.20**	**0.31**	**0.49**	0.71	**0.15**
State of the art VINS [24]	0.26	0.52	0.79	**0.62**	0.20

## Supplementary Materials

The following are available online at www.mdpi.com/1424-8220/17/12/2795/s1, Video Traj5.mp4 shows the real time results of MI-MSCKF filter on Traj5 of the dataset.
